# Comment on: Assessing ChatGPT’s ability to answer questions pertaining to erectile dysfunction

**DOI:** 10.1038/s41443-023-00821-2

**Published:** 2024-03-11

**Authors:** Jacob S. Hershenhouse, Giovanni E. Cacciamani

**Affiliations:** 1 USC Institute of Urology and Catherine and Joseph Aresty Department of Urology, Keck School of Medicine, University of Southern California, Los Angeles, CA USA; 2 Artificial Intelligence Center, USC Institute of Urology, University of Southern California, Los Angeles, CA USA

**Keywords:** Sexual dysfunction, Quality of life

Due to the sensitive and sexual nature of the condition, many men opt to consult the internet first when seeking to understand Erectile Dysfunction (ED), anonymously researching the subject and any potential solutions online [1] prior to enlisting their primary care physician or urologist for advice and treatment. Now with the rapid release of publicly accessible, consumer-facing Generative Artificial Intelligence (GAI) like ChatGPT, studying the efficacy of patient-facing GAI is crucial as the technology evolves. We have read with interest the recent study by Razdan et al., which raises important questions about the capabilities and limitations of large language models like ChatGPT applied to answering common inquiries about ED [2]. A number of significant conclusions can be drawn from their results.

It is crucial to recognize that GPT models are not specifically trained in medical knowledge, unlike specialized systems such as Medpalm2 [3]. Despite GPT’s impressive ability to respond to medical inquiries, there is an inherent risk of inaccuracies when addressing public questions. A significant concern is the manner in which these chatbots deliver responses. The “human-like” format and style of their outputs can mislead patients into over-trusting this “AI oracle” without skepticism. This is particularly perilous because, unlike Google searches where users actively select from numerous links or webpages, the sources behind AI-powered chatbot responses are obscured, and the output is singular. This raises an essential issue: as physicians, we must engage actively in the development and evaluation of these AI-chatbots, rather than passively accept them or become involved only at the final stages. In the business of medicine, which fundamentally relies on trust, the accuracy and validation of the knowledge used by GPTs in responding to patient inquiries must be meticulously scrutinized and validated [4].

We found the results of the authors’ readability analysis particularly telling and generalizable. Prior studies have shown that ChatGPT produces (when not otherwise prompted) standard outputs of post-secondary grade level when employed for various medical use cases [5, 6]. We agree with the authors that the reading level and terminology used by ChatGPT exceeds the health literacy level of many ED patients. It is best practice to not exceed a Flesch-Kincaid Grade Level readability score above grade level 8 on medical documentation intended for patient understanding, as is the case for informed consent forms [7]. It is the role of the physician to educate the patient about finding verifiable, understandable information online for their conditions [8]. Simply put, the more patients truly comprehend, the better. To improve the readability of generated outputs, and therefore the level of understanding of the online patient population, we believe that prompting the chatbot to simplify its explanations and use more colloquial terms could make ChatGPT’s responses more accessible when the responses are intended specifically to be patient-facing.

We also found their interpretation of ChatGPT’s “empathy” to merit further investigation. Men’s health conditions necessitate empathetic answers from healthcare professionals and source material, which extends inherently to online resources. ChatGPT’s ability to demonstrate empathy and provide couples counseling reveals the technology’s potential to aid in reducing the stigma around ED and encourage open communication on the subject before one comes into clinic. This ability to incorporate supportive language suggests this technology could be useful in co-piloting, alongside healthcare professionals, online information seeking particularly for men’s health diseases.

**Fig. 1:**
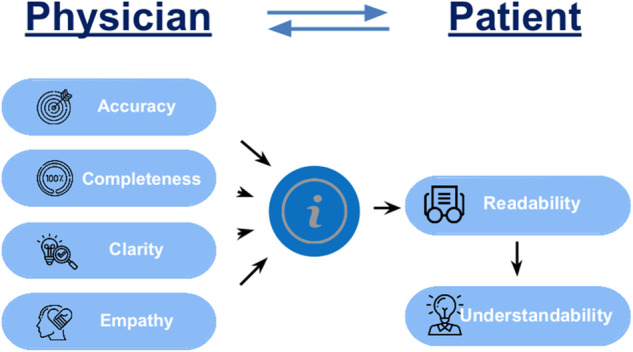
Keyfeatures for assessing AI-powered medical chatbots response quality.

In response to the subjective interpretation of response quality, we caution against overgeneralizing ChatGPT’s lack of nuance regarding treatment modalities as proof it is incapable or untrustworthy in a medical context. OpenAI’s usage policies warn against this potential use case for the software: “You should never use our models to provide diagnostic or treatment services for serious medical conditions [9].” Despite this warning, the software does still attempt to provide this information, though its knowledge base is restricted to before Q4 2021, which leads to an expected dip in performance when assessed by up-to-date, expert providers [10–12]. These results underscore the need for greater fine-tuning of these models if they are to fit this use case in patient education and we join other researchers in calling for greater regulation of this software in all medical applications [13–15]. Medical researchers conducting exploratory research into other medical use cases should heed the usage policies for the software under examination as well as ethical concerns surrounding the technology as well. With this in mind, it begs the question: why is there no standardized method to ascertain the quality of chatbot generated responses to medical questions? In an effort to allow researchers to compare and replicate studies such as this one, we propose the following 5-item quantitative analysis for assessing response quality: Accuracy, Completeness, Clarity, Readability, and Understandability and empathy (see Fig. 1). Without assessing each of these essential reflections of quality, the validity of conclusions based in an incomplete research methodology in this domain could be called into question.

While an imperfect source of ED information for patients, ChatGPT demonstrates the promise of AI to supplement human clinical expertise and disseminate medical information if employed correctly. Yet, the information provided, even when generated by machine, should be always supported by clinical evidence and reference. With appropriate oversight and updating as the technology evolves, large language models could meaningfully expand access to consumer health education on sexual health. More interdisciplinary research is needed, but our shared goal remains empowering patients with accurate, empathetic ED knowledge.
